# *Psychrobacter* Infections in Humans—A Narrative Review of Reported Cases

**DOI:** 10.3390/antibiotics14020140

**Published:** 2025-02-01

**Authors:** Petros Ioannou, Afroditi Ziogou, Alexios Giannakodimos, Ilias Giannakodimos, Andreas G. Tsantes, George Samonis

**Affiliations:** 1 Department of Internal Medicine, University Hospital of Heraklion, 71110 Heraklion, Greece; 2 School of Medicine, University of Crete, 71003 Heraklion, Greece; 3 Department of Medical Oncology, Metaxa Cancer Hospital of Piraeus, 18537 Piraeus, Greece; 4 Department of Cardiology, Tzaneio General Hospital of Piraeus, 18537 Piraeus, Greece; 5 Department of Urology, Attikon General Hospital of Athens, 12462 Athens, Greece; 6 Laboratory of Hematology and Blood Bank Unit, “Attikon” University Hospital, School of Medicine, National and Kapodistrian University of Athens, 12462 Athens, Greece; andreas.tsantes@yahoo.com; 7 First Oncology Department, Metropolitan Hospital, 18547 Neon Faliron, Greece

**Keywords:** *Psychrobacter*, infection, bacteremia, pneumonia, endocarditis, osteomyelitis, peritonitis

## Abstract

Background: *Psychrobacter* species are aerobic, Gram-negative, spherical-to-rod-shaped, psychrophilic bacteria that belong to the *Moraxellaceae* family. In spite of their uncommon manifestation in the general population, infections due to *Psychrobacter* spp. are increasingly identified especially in immunocompromised individuals or patients with severe comorbidities. Objectives: This review aims to analyze all reported instances of *Psychrobacter* spp. infections in humans, with an emphasis on data pertaining to epidemiology, microbiology, antimicrobial resistance, treatment strategies, and mortality outcomes. Methods: A narrative review was performed through a literature search of PubMed/MedLine and Scopus databases. Results: In total, 12 articles offered data on 12 patients infected with *Psychrobacter* spp. Their mean age was 33.41 years, while 63.64% of them were male. Immunosuppression was the predominant risk factor (33.3%). Bacteremia was the most commonly observed type of infection (41.6%), followed by meningitis, skin infection, and conjunctivitis. *Psychrobacter immobilis* was the most usually identified species (33.3%). The pathogen exhibited sensitivity to most antimicrobials. The most widely administered antimicrobials included cephalosporins (70%), followed by aminopenicillins and vancomycin (40%, respectively). The clinical outcome depended primarily on the infection site; mortality rate was high (44.4%), especially in cases of bacteremia (50%). Conclusion: Due to the potential of *Psychrobacter* spp. to cause serious infection, clinicians and laboratory professionals should consider it in the differential diagnosis in patients with infections by Gram-negative spherical bacteria, particularly in patients with significant comorbidities and immunodeficiency, in order to accurately establish the diagnosis and proceed to the right treatment.

## 1. Introduction

*Psychrobacter* species are Gram-negative, aerobic bacteria belonging to the *Moraxellaceae* family and known for their ability to tolerate cold temperatures (psychrotrophic) [1]. These microorganisms have been identified in various sources, including marine species such as fish, crustaceans, and marine mammals, as well as marine environments like seaweed and the seabed. They have also been isolated from food products such as cheese, seafood, and meat, and animals like storks and pigs (specifically from their digestive tracts) and lamb lungs [2]. *Psychrobacter* spp. may be part of the normal human microbiota; certain studies detected species such as *P. arenosus*, *P. faecalis*, *P. phenylpyruvicus*, and *P. pulmonis* in the human gut microflora [3]. Human infection due to *Psychrobacter* spp. is exceedingly rare; only a small number of *Psychrobacter* species are regarded as medically significant opportunistic pathogens, based on a limited collection of published case studies [4]. *Psychrobacter* has been linked to wound infections associated with exposure to oceanic or marine environments, as well as several bacteremia and meningitis cases [5]. The identification of *Psychrobacter* spp. is considered particularly challenging, often leading to misidentification with genera such as *Neisseria* due to their phenotypical similarities [6]. This misidentification may result in a global underdiagnosis of *Psychrobacter* infections. Accurate identification relies on advanced microbiological methods, such as 16S rRNA gene sequencing [7]. Currently, no standardized treatment guidelines exist for *Psychrobacter* infections and management typically involves empirical therapy while awaiting antimicrobial susceptibility results. Mortality rates are relatively high, with outcomes largely dependent on the site of infection and the patient’s underlying health status.

The present review aimed to review all documented cases of *Psychrobacter* spp. infections in humans, with a focus on epidemiology and mortality data. It also sought to explore the microbiology, antimicrobial susceptibility, and treatment strategy of these infections. Additionally, another goal was to address gaps in knowledge, such as risk factors and therapeutic approaches, and to expand the limited literature on this emerging pathogen.

## 2. Results

### 2.1. Characteristics of Included Studies

**Figure 1:**
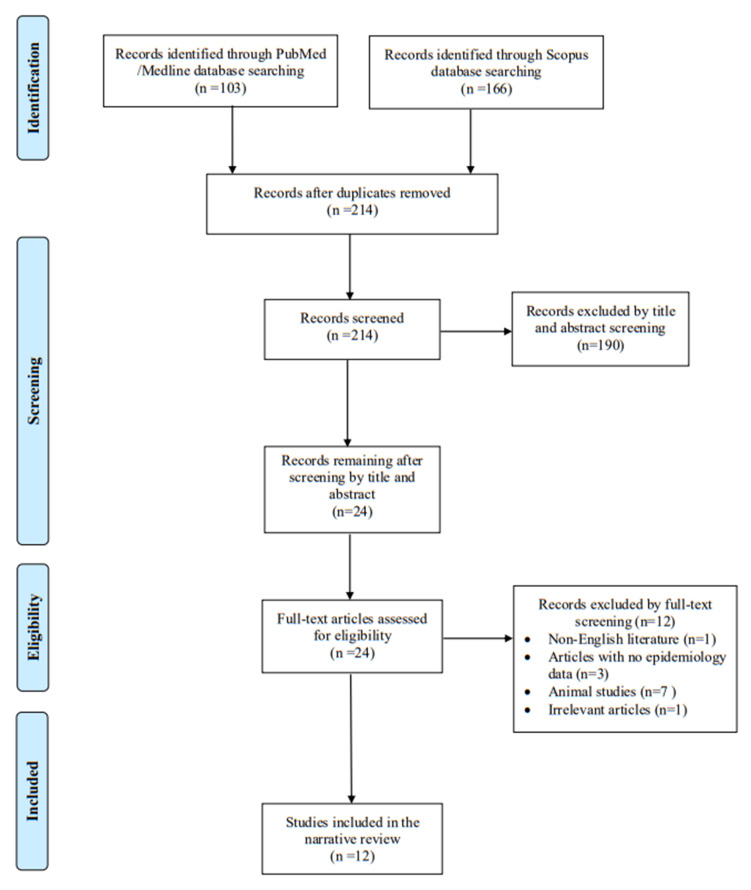
Trial flow of this narrative review.

**Figure 2:**
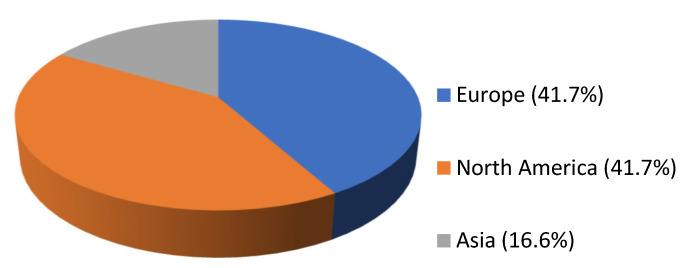
Pie chart of geographical distribution of *Psychrobacter* spp. infections globally.

A total of 269 articles were screened from PubMed and Scopus databases. Eventually, after duplicate removal, record screening, and applying the snowball procedure, only 12 articles met the inclusion criteria and were selected for analysis [2,5,6,7,8,9,10,11,12,13,14,15]. These studies presented data on 12 patients in total. A flow diagram of the selection process is illustrated in Figure 1. Among the included cases, 5 occurred in Europe (41.7%), 5 in North America (41.7%), and 2 in Asia (16.6%). All articles selected for the present review were case reports. Figure 2 represents the geographical distribution of all *Psychrobacter* spp. infections worldwide. Supplementary Table S1 shows the characteristics of the included studies in the present review.

### 2.2. Epidemiology of Psychrobacter spp. Infections

The mean age of patients with *Psychrobacter* spp. infections was 33.41 years, varying from 0 to 67 years, while 63.64% (7 patients) were male. Regarding patients’ medical history and predisposing risk factors, four patients (33.3%) were immunosuppressed, three (25%) were previously diagnosed with lung disease, while two (16.6%) who underwent recent surgical procedures had a ventriculoperitoneal shunt, external ventricular drain, or a history of premature birth. Only one patient (8.3%) had a central venous catheter (CVC), while one other patient (8.3%) was an HIV-positive intravenous drug user. The demographic and clinical characteristics of *Psychrobacter* spp. cases are depicted in Table 1.

### 2.3. Antimicrobial Resistance and Microbiology of Psychrobacter spp. Infections

*Psychrobacter* spp. was isolated from blood cultures in six patients (50%) and from cerebrospinal fluid (CSF) in four (33.3%), while in one patient (8.3%), the pathogen was detected in both of these biological specimens. Notably, in three patients (25%), the pathogen was isolated from wound or pus cultures. *Psychrobacter immobilis* was the most usually identified species (33.3%), followed by *Psychrobacter sanguinis* (25%) and *Psychrobacter phenylpyruvicus* (16.6%). In one case, the pathogen was identified as *Psychrobacter piechaudii* and, in one other patient, as *Psychrobacter arenosus.* Only one patient (8.3%) developed polymicrobial infection, with *Staphylococcus* spp. being the coexisting microorganism detected. Pathogen identification was accomplished with advanced genetic testing. More precisely, in the majority of cases (77.7%), 16s-rRNA sequencing managed to identify the pathogen. VITEK was also successfully applied in one patient (11.1%). In regard to antimicrobial resistance, the disk diffusion method was most commonly performed in eight patients (88.8%). The antimicrobial resistance of *Psychrobacter* spp. is shown in Table 2.

### 2.4. Clinical Presentation of Psychrobacter spp. Infections

*Psychrobacter* spp. infections most commonly involved the bloodstream (in 5 patients; 41.6%) and the central nervous system (CNS) (in 4 patients; 33.3%). Skin infections were noticed in two patients (16.7%), while only one patient (8.3%) developed conjunctivitis. The duration of symptoms ranged between 0 (acute onset) and 31 days.

### 2.5. Treatment and Outcome of Psychrobacter Infections

Based on the available data, 10 patients received antimicrobial treatment. Cephalosporins were the most frequently administered antimicrobial agents (in 7 patients), followed by aminopenicillins and vancomycin (in 4 patients). Three patients received carbapenems, while two received either quinolones or aminoglycosides. Of note, carbenicillin, antistaphylococcal penicillin, piperacillin/tazobactam, fosfomycin, macrolides, teicoplanin, and metronidazole were rarely used, each of them in one patient. Concerning the remaining patients, the therapeutic approach was not mentioned in the text of the original case reports. Surgical interventions were applied in combination with antimicrobial treatment in 2 out of 12 patients. The median treatment duration for survivors was 14 days. The overall mortality rate was estimated at 44.9% (4 out of 9 patients), while the mortality rate associated with *Psychrobacter* spp. infection was specifically 33.3% (3 out of 9 patients). In one patient, the cause of death was underlying cardiovascular disease.

### 2.6. Bacteremia Due to Psychrobacter Infection

Bloodstream infection was detected in five patients. Regarding this patient group, the mean age was 46.2 years and four patients were male. Predisposing risk factors involved immunosuppression in four patients; in particular, one patient was HIV-positive, while two different patients had a history of grade 3 myelofibrosis and X-linked chronic granulomatous disease. One patient had a central venous catheter (CVC) and another one had received antimicrobials within the last three months. Interestingly, one patient had recently consumed raw geoduck clam. All patients with *Psychrobacter* bacteremia developed fever, three patients developed systemic inflammatory response syndrome (SIRS), while two presented with lung dysfunction and kidney failure. Of note, septic shock was observed in one and seizures in another patient. The mean treatment duration was 11 days. Antimicrobial agents were administered to 4 patients and most commonly involved aminopenicillins, cephalosporins, and vancomycin in 2 out of 4 based on the available data. Overall mortality was calculated at 50% (2 out of 4 patients, with the available data), while mortality attributed to *Psychrobacter* infection was also specifically 50%.

### 2.7. CNS Infection Due to Psychrobacter

Infection located in the CNS was observed in four patients. Among these patients, 50% were male and the mean age was 38.5 years; interestingly, two patients were neonates. Two patients had ventriculoperitoneal shunts or external ventricular drains, one was born prematurely and was diagnosed with lung disease and twin-to-twin transfusion syndrome, while the other patient had undergone a surgical procedure within the last three months. None of these patients had a medical history of immunosuppression. Fever and organ dysfunction were present in three patients, while renal failure, loss of consciousness, and SIRS were observed each in one patient. In this patient group, the mean antimicrobial treatment was 19 days; cephalosporins constituted the most widely used antimicrobials in three patients. The overall mortality rate and mortality due to *Psychrobacter* infection were 33.3% (1 out of 3 patients with the available data).

### 2.8. Other Infections Due to Psychrobacter spp.

Skin infection was diagnosed only in two patients. One was male and the mean age of this patient group was 46.5 years. One patient had recently undergone surgery and had a history of pulmonary hypertension, aortomitral valve insufficiency, and arrhythmia. Regarding the second patient, marine exposure was the only predisposing factor reported. One patient developed fever, heart failure, and embolic phenomena in the right common iliac and right femoral artery. The second patient did not develop any complications. Both patients received cephalosporins, while one of them additionally received vancomycin and carbapenem and underwent wound drainage. The overall mortality rate was estimated at 50% (one patient), while no deaths were associated with *Psychrobacter* infection exclusively. Finally, conjunctivitis was reported in one patient, who was a neonate of premature birth, diagnosed with grade 2 hyaline membrane syndrome and congenital syphilis. No data on clinical outcome were available regarding this patient.

## 3. Discussion

This review examines the features of infections caused by *Psychrobacter* species, collecting information from multiple studies that provided comprehensive insights about their epidemiology, microbiology, clinical manifestations, treatment strategies, and outcomes. Infection of the bloodstream and the CNS was the most frequently documented, while skin infection and conjunctivitis were also reported. Among the identified species, *Psychrobacter immobilis* was the most prevalent. Cephalosporins were the most commonly used antimicrobial agents for treatment. The review also emphasizes the notably high overall mortality rate associated with *Psychrobacter* infections.

The scarcity of reported cases of *Psychrobacter* spp. infections in the existing literature renders the establishment of accurate epidemiological data for these infections highly demanding [8]. In this review, the majority of cases occurred in male patients, with a median age of 28 years. Interestingly, an equal number of cases was reported in European and North American countries, while only two cases were identified in Asia. The higher prevalence in Europe and North America could be linked to more robust clinical surveillance systems in these geographical areas. Conversely, the lower prevalence in Asia, despite the pathogen’s isolation from animal species and the absence of reported cases in Africa, suggests that *Psychrobacter* infections may not be strongly associated with poor living conditions [16]. However, the reduced number of studies worldwide makes it challenging to draw definitive epidemiological conclusions about *Psychrobacter* spp. infections.

The genus *Psychrobacter* belongs to the *Moraxellaceae* family, alongside other genera such as *Moraxella* and *Acinetobacter*. The genus *Psychrobacter* was established in 1986.These bacteria are strictly aerobic, Gram-negative, non-motile, catalase-positive, spherical-to-rod-shaped, psychrophilic organisms, capable of thriving and reproducing at temperatures below 10 °C. Currently, 57 species within the *Psychrobacter* genus have been documented [8,17]. Due to its extremophilic nature, *Psychrobacter* is commonly linked to Antarctic and marine ecosystems [2]. Numerous other *Psychrobacter* species have been identified, primarily from environmental sources and poikilothermic animals. However, two species, *Psychrobacter faecalis* and *Psychrobacter pulmonis*, were lately discovered in warm-blooded animals, specifically in pigeon feces [18]. These bacteria may also be a part of the human microbiota; studies have shown the presence of *P. arenosus*, *P. faecalis*, *P. phenylpyruvicus*, and *P. pulmonis* in the human gut [3]. More recently, clinical isolates have been identified and belong to a newly described species: *Psychrobacter sanguinis* [19]. *Psychrobacter* species are regarded as rare opportunistic pathogens in humans and have been detected in various clinical samples, including the brain tissue, urine, ears, eyes, vulvae, wounds, cerebrospinal fluid (CSF), and blood [4,14,19]. Of note, the majority of these isolates are classified as *Psychrobacter faecalis* and *Psychrobacter pulmonis* [4]. In spite of the pathogen’s detection in human samples, due to the limited number of published studies and data available on these pathogens, their true clinical significance in human infections remains largely unclear.

Although *Psychrobacter* species are typically regarded as opportunistic pathogens, several virulence genes commonly associated with pathogenic bacteria have been detected in these species, including genes involved in antimicrobial resistance, cell invasion, the type IV secretion system, and iron uptake [7]. The *OmpA* gene, which codes for an outer membrane protein, plays a key role in the invasion of brain microvascular endothelial cells, a crucial step for successfully crossing the blood–brain barrier [20]. Another virulence factor revealed in *Psychrobacter* spp. is a heme ABC transporter; the ATP-binding cassette (ABC) transporters are a large family of proteins with a highly conserved ATPase domain that binds and hydrolyzes ATP. These transporters are responsible for importing and exporting a variety of substrates and are essential for bacteria to uptake vital nutrients, such as iron [7,21]. Finally, genes encoding the type IV secretion system have been discovered in *Psychrobacter* spp. Type IV pilins (T4P) are structural proteins that play a role in various processes, including surface adhesion, aggregation, and DNA uptake and twitching motility [22].

Regarding potential predisposing risk factors, a history of immunosuppression emerged as the most common, based on the findings of this review. In recent years, the incidence of infections caused by uncommon pathogens in immunosuppressed individuals has significantly increased [23]. Notably, one case with *Psychrobacter* bacteremia was diagnosed in an HIV-positive patient. HIV-infected patients exhibit a higher prevalence of bacteremia when compared to the general population [24]. Especially in cases of advanced stages of AIDS, these infections manifest with greater seriousness and may lead to septic shock, a poor response to antimicrobial treatment, and elevated mortality rates [25]. Other causes of immunosuppression documented in the present review constitute the X-linked chronic granulomatous disease (CGD) and the advanced-stage myelofibrosis [5,12]. CGD is a primary disorder of phagocytic cells caused by mutations in one of five subunits that make up the phagocyte NADPH oxidase complex. These mutations result in reduced superoxide production, compromising the intracellular killing of specific bacteria and fungi. Consequently, individuals with CGD are particularly vulnerable to a limited range of catalase-positive organisms, such as *Psychrobacter* spp. [26]. In these patients, septicemia is less frequent but can be life-threatening when it occurs; similarly, the patient included in this review developed septicemia, multiple organ failure, and eventually died [12].Moreover, another case of immunosuppression involved hepatitis C and liver cirrhosis [13]. Patients with cirrhosis experience an acquired immune deficiency caused by various factors, including dyshomeostasis and malnutrition. This condition impairs multiple host defense mechanisms, such as the acute phase response and the functions of macrophages, neutrophils, and lymphocytes [27]. Additionally, in individuals with portal hypertension, the liver’s ability to filter bacteria may be bypassed, leading to a more than tenfold increase in the incidence of bacteremia [28].

Another important risk factor noticed in the included patients was lung disease, as well as recent surgical procedures. Lung disease mainly involved chronic pulmonary pathologies, pulmonary hypertension, and grade 2 hyaline membrane syndrome [6,8,9]. Recent surgical procedures, within the previous three months, may be complicated with wound infection by *Psychrobacter*, presenting with fever and leucocytosis [9]. Additionally, two of the included cases had a ventriculoperitoneal shunt or external ventricular drain and developed post-neurosurgical meningitis [8,11]. Investigations were conducted to determine the source of *Psychrobacter* spp., but it remained unclear how this bacterium caused post-neurosurgical meningitis. One possible explanation is that the pathogen was present in the patient’s environment, possibly prior to hospitalization, and penetrated the central nervous system through colonization of the external ventricular drain. Alternatively, the pathogen may have penetrated the CNS through the bloodstream, although this is considered less probable [11]. It has also been speculated that *Psychrobacter* spp. can form biofilms on prosthetic material, such as VP shunts [8]. Interestingly, two patients were exposed to the marine environment prior to development of the infection. A study by Bonwitt et al. describes a wound infection caused by *Psychrobacter* spp. in a healthy individual, after exposure to squid bait and seawater, while a study by Leung et al. presents a case of *Psychrobacter* bacteremia in a patient that consumed raw geoduck clam. Data from these studies suggest that the infection may have resulted from exposure to the marine environment even in cases without serious comorbidities [2,13].

Identifying *Psychrobacter* species remains complicated, as most microbiology laboratories lack access to innovative molecular techniques, such as genetic testing. Moreover, the detection of non-fermentative Gram-negative bacilli such as *Psychrobacter* spp. may be challenging, partly since certain taxa are excluded from the databases of most commercial systems, and partly because some species are non-reactive or show slow growth [29]. Misidentification with *Neisseria* species is common due to their overlapping phenotypes [6]. Therefore, maintaining high clinical and microbiological suspicion is crucial for prompt and accurate identification. Advanced microbiological techniques, such as 16S rRNA sequencing or PCR techniques, are typically required for more accurate identification. In this review, 16s rRNA was the most frequently applied method for *Psychrobacter* identification. In a study by Ortiz-Alcantara et al., next-generation sequencing (NGS) has also been used for metagenomic analysis to identify the pathogen [7]. NGS technologies have made significant progress in recent years, providing the ability to detect pathogens by identifying small amounts of DNA or RNA sequences at a lower cost [7,30].The total DNA collection from biological fluids can aid in the isolation of specific *Psychrobacter* spp. sequences. While whole-genome sequencing through shotgun metagenomics is unlikely, it may serve as a powerful culture-independent method for pathogen identification [7]. The use of NGS in *Psychrobacter* detection has not been thoroughly investigated in the literature and further studies are required to collect relevant data. Due to increased cost and limited availability of these techniques, accurate diagnosis should also depend on the biochemical or cultural characteristics specific to the pathogen. Conventional bacterial cultures were conducted in biological specimens of all included patients and provided biochemical and cultural data. A positive bacterial culture may constitute the primary laboratory finding that generates further innovative and advanced molecular methods.

To date, no official or widely recognized antimicrobial susceptibility breakpoints have been established for *Psychrobacter* spp., as formal studies evaluating its susceptibility profile are lacking. Consequently, the data presented in this review rely on case reports that provide information on the pathogen’s antimicrobial susceptibility. Previous studies indicated susceptibility to antimicrobials such as cefotaxime, ceftazidime, piperacillin, amikacin, ciprofloxacin or cephalothin, gentamicin, chloramphenicol, and erythromycin [6,10,14]. However, the sensitivity to meropenem and trimethoprim-sulfamethoxazole has not yet been described. In general, *Psychrobacter* spp. strains exhibit high susceptibility to various antimicrobial drugs [5]. In the present review, resistance to vancomycin, penicillin, aminopenicillins, and macrolides was observed in only a few cases. Resistance to macrolides has been associated with genes encoding a Zn-dependent metallo-beta-lactamase and macrolide-specific ABC-type efflux-related proteins, present in *Psychrobacter* spp. An ATP-binding permease and a peripheral membrane protein belonging to the membrane fusion protein family have been reported to induce macrolide resistance [7,31].As no precise explanation has been provided for the exact resistance patterns of these pathogens, further investigations into these resistance mechanisms are necessary. Additionally, establishing specific criteria for interpreting sensitivity assays remains crucial.

The management of *Psychrobacter* spp. infections remains particularly challenging, considering that no official antimicrobial administration guidelines have been established. Aggressive antimicrobial treatment is recommended in cases of systemic infection to prevent a potentially fatal outcome [10]. Most human infections have been successfully treated with third-generation cephalosporins, resulting in rapid recovery [13]. Based on the data from this review, cephalosporins appear to be the optimal treatment for severe *Psychrobacter* infections, as the pathogens were susceptible to cephalosporins in most cases. Aminopenicillins and vancomycin were frequently used to treat *Psychrobacter*-infected patients, followed by carbapenems, quinolones, and aminoglycosides. Antimicrobial treatment should be guided by in vitro susceptibility testing to ensure optimal treatment response. The duration of treatment depends on the infection site and the intensity of symptoms. In this review, treatment duration ranged from 4 to 28 daysin cases of severe complications, such as organ failure [8]. Surgical procedures were applied in two cases, alongside antimicrobial treatment, and included surgical wound drainage and the removal of a previous shunt and temporary external ventricular drain followed by new VP shunt insertion [8,9]. Mortality rates associated with *Psychrobacter* infections are elevated, particularly among patients with severe comorbidities or those who develop complications like sepsis or multi-organ failure [10]. This increased mortality can often be linked to delayed or incorrect diagnosis, especially when the infection is mistaken for other, similar bacteria. Additionally, the pathogen’s ability to cause severe infections, the challenge in accessing accurate diagnostic tools, and its unknown resistance mechanisms may further contribute to the high mortality rate.

This study has some notable limitations. The literature search may not have included all relevant studies on epidemiology and mortality, and some studies could have been missed due to the search strategy. Our analysis was based solely on case reports and case series, which depend on accurate record-keeping to ensure credibility. Additionally, many studies did not employ molecular identification methods, such as 16S rRNA sequencing, which increases the risk of misidentification in some cases. Furthermore, some studies presented incomplete data, which restricted the scope of our statistical analysis to the information available. Consequently, we could only present findings from studies that provided complete data. Finally, the exclusion of studies in languages other than English could introduce a potential sample bias; however, the number of such articles was minimal.

## 4. Materials and Methods

### 4.1. Search Strategy and Inclusion and Exclusion Criteria

This narrative review aims to collect and present all published data on *Psychrobacter* species infections in humans. The primary objective was to present data on epidemiology and mortality rate. Secondary outcomes focused on data regarding the specific infection site, clinical characteristics of the included patients, microbiological features, and treatment options. For this review, PubMed/Medline and Scopus databases were searched for potential articles addressing all *Psychrobacter* spp. infections by two independent investigators (A.Z. and A.G.) starting on October 1st until November 5th, 2024. Data were gathered using a predefined template. The following keywords were applied for the search strategy: “*Psychrobacter*” AND (“infection” OR “bacteremia” OR “endocarditis” OR “peritonitis” OR “pneumonia” OR “osteomyelitis”).Any disputes that arose were settled through the involvement of a senior investigator (P.I.). The inclusion criteria for this review encompassed studies presenting original data, such as case series, case reports, and cohort studies, which offered insights into the epidemiology and clinical outcomes of *Psychrobacter* spp. infections in humans. Only studies published in the English language were considered. Reviews and systematic reviews with cumulative data were excluded. Studies involving animals, articles without full-text access, or those lacking adequate information on patients’ mortality and epidemiology were also not considered eligible. To ensure thorough coverage, the references of all included articles were examined to identify any studies that were potentially missed in the initial search.

### 4.2. Data Extraction and Definitions

The data gathered from each selected study included publication year, article type, country of origin, patient demographics (age, gender), relevant medical history, details of infection, and key clinical characteristics, such as specific infection site and complications, as well as microbiological characteristics, such as identified pathogen, antibiotic susceptibilities, and, finally, treatment regimens used and outcome (survival or mortality). The relationship between mortality and the initial infection was documented according to each study’s authors.

### 4.3. Statistical Analysis

Data are presented as numbers (%) for categorical variables and median (interquartile range, IQR) for continuous variables. Continuous variables were compared using the Mann–Whitney U-test for non-normally distributed variables or the t-test for normally distributed variables. All tests were two-tailed, and a p-value equal to or lower than 0.05 was considered significant. Statistics were calculated with GraphPad Prism 6.0 (GraphPad Software, Inc., San Diego, CA, USA).

## 5. Conclusions

This review provides valuable insights into the epidemiology, clinical features, antimicrobial susceptibility, microbiology, treatment, and outcomes of *Psychrobacter* infections, emphasizing key information on the pathogenic potential of this microorganism. *P. immobilis* was the most commonly identified species, and the bloodstream was most regularly affected. The pathogen exhibited sensitivity to most antimicrobial agents. Even though official therapeutic guidelines are lacking, cephalosporins were the most commonly used antimicrobials. The site of infection plays a critical role in the disease’s outcome, with each clinical severity being closely related to the patient’s immune state. Antimicrobial treatment should start promptly and be guided by in vitro susceptibility testing. Due to the opportunistic potential of *Psychrobacter* spp. to cause serious infection and the challenges posed by commercial identification systems, healthcare providers and laboratory professionals should be aware of these rare pathogens to accurately confirm the diagnosis. Despite the limitations of this review, it may encourage further longitudinal research and controlled studies to better understand *Psychrobacter* infections and provide essential information for future treatment strategies.

## Figures and Tables

**Table 1:** Characteristics of patients with *Psychrobacter* species infection.

Characteristic	All Patients (*n* = 12) *	Survived (*n* = 5) *	Died (*n* = 4) *
Age, years, median (IQR)	28 (3.3–63.5)	58(13–63)	23 (13.8–57.8)
Male gender, *n* (%)	7 (63.64)	3 (60)	3 (75)
Predisposing factors			
Postsurgery, *n* (%)	2 (16.67)	1 (20)	1 (25)
Lung disease, *n* (%)	3 (25)	1 (20)	1 (25)
IVDU, *n* (%)	1 (8.33)	0	1 (25)
Immunosuppression, *n* (%)	4 (33.33)	2 (40)	2 (50)
HIV, *n* (%)	1 (8.33)	0	1 (25)
Central venous catheter, *n* (%)	1 (8.33)	0	1 (25)
VP shunt or external ventricular drain, *n* (%)	2 (16.67)	2 (40)	0
Bacteremia, *n* (%)	5 (41.67)	2 (40)	2 (50)
CNS infection, *n* (%)	4 (33.3)	2 (40)	1 (25)
Polymicrobial infection, *n* (%)	1 (8.33)	1 (20)	0
Clinical characteristics			
Fever, *n* (%)	9/12 (75)	3 (60)	4 (100)
Sepsis, *n* (%)	4/12 (33.33)	1 (20)	3 (75)
Treatment			
Cephalosporin, *n* (%)	7/10 (70)	3 (60)	3 (75)
Aminopenicillin, *n* (%)	4/10 (40)	3 (60)	0
Vancomycin, *n* (%)	4/10 (40)	1 (20)	3 (75)
Carbapenem, *n* (%)	3/10 (30)	1 (20)	2 (50)
Quinolone, *n* (%)	2/10 (20)	1 (20)	1 (25)
Outcomes			
Deaths due to infection, n (%)	3/9 (33.3)	NA	NA
Deaths overall, n (%)	4/9 (44.4)	NA	NA

CNS: central nervous system; HIV: human immunodeficiency virus; IQR: interquartile range; NA: not applicable; VP: ventriculoperitoneal shunt; * data are among the number of patients mentioned on top unless otherwise described.

**Table 2:** Antimicrobial resistance rates.

Antimicrobial Agent	Number of Patients	Resistance (%)
Vancomycin	2/4	50
Penicillin	2/5	40
Macrolides	1/4	25
Aminopenicillins	1/6	16.67

## Data Availability

Not applicable.

## Supplementary Materials

The following supporting information can be downloaded at https://www.mdpi.com/article/10.3390/antibiotics14020140/s1, Table S1: Characteristics of all included studies.
