# Supplementary material for: Psychrobacter Infections in Humans—A Narrative Review of Reported Cases
Source: Antibiotics (Basel). 2025 Feb 1;14(2):140. doi: 10.3390/antibiotics14020140 (PMC11851457; doi:10.3390/antibiotics14020140)
Supplement: Supplementary file 1 [file antibiotics-14-00140-s001.zip › antibiotics-3432924-supplementary.pdf]

Author, Year	Number of Patients	Gender	Age (Years)	Site of Infection (%)	Treatment (%)	Mortality (%)
Kumaria et al., 2021 [8]	1	Female	0	CNS (100)	Cephalosporin, Teicoplanin, Quinolone, Aminoglycoside (100)	0 (0)
Bonwitt et al., 2018 [2]	1	Male	26	Skin (100)	Cephalosporin (100)	0 (0)
Ortiz-Alcantara et al., 2016 [7]	1	Male	13	CNS (100)	Cephalosporin, Piperacilllin/ Tazobactam, Vancomycin, Macrolide (100)	1 (100)
Stepanovic et al., 2007 [9]	1	Female	67	Skin (100)	Cephalosporin, Carbapenem, Vancomycin (100)	1 (100)
Lozano et al., 1994 [10]	1	Male	30	Bacteremia (100)	Cephalosporin, Quinolone (100)	1 (100)
Le Guern et al., 2014 [11]	1	Female	64	CNS (100)	Aminopenicillin, Carbapenem, Fosfomycin (100)	0 (0)
Caspar et al., 2013 [5]	1	Male	58	Bacteremia (100)	Aminopenicillin, Anti-staphylococcal penicillin, Carbenicillin, Vancomycin (100)	0 (0)
Gini et al., 1990 [6]	1	NR	0	Conjunctivitis (100)	NR	NR
Sriaroon et al., 2014 [12]	1	Male	16	Bacteremia (100)	Carbapenem, Vancomycin (100)	1 (100)
Leung et al., 2005 [13]	1	Male	62	Bacteremia (100)	Aminopenicillin, Cephalosporin, Metronidazole (100)	0 (0)
Lloyd-Puryear et al., 1991 [14]	1	Male	0	CNS (100)	Aminopenicillin, Cephalosporin, Aminoglycoside (100)	NR
Nuha et al., 2020 [15]	1	Female	65	Bacteremia (100)	NR	NR

Supplementary Table S1. Characteristics of all included studies

CNS: Central nervous system, NR: not reported
